# *Escherichia coli* alanyl-tRNA synthetase maintains proofreading activity and translational accuracy under oxidative stress

**DOI:** 10.1016/j.jbc.2022.101601

**Published:** 2022-01-20

**Authors:** Arundhati Kavoor, Paul Kelly, Michael Ibba

**Affiliations:** 1 The Ohio State University Molecular, Cellular and Developmental Biology Program, The Ohio State University, Columbus, Ohio, USA; 2 Center for RNA Biology, The Ohio State University, Columbus, Ohio, USA; 3 Department of Microbiology, The Ohio State University, Columbus, Ohio, USA; 4 Schmid College of Science and Technology, Chapman University, Orange, California, USA

**Keywords:** aminoacyl-tRNA synthetases, alanyl-tRNA synthetase, transfer RNA, oxidative stress, translational control, aaRS, aminoacyl-tRNA synthetase, AlaRS, alanyl-tRNA synthetase, Cys-SOH, cysteine sulfenic acid, DNPH, 2,4-dinitrophenylhydrazine, DTNB, thio-bis-(2-nitrobenzoic acid), Gn-HCl, guanidine hydrochloride, H_2_O_2_, hydrogen peroxide, MetRS, methionyl-tRNA synthetase, Met-SO, methionine sulfoxide, MS, mass spectrometry, PheRS, phenylalanyl-tRNA synthetase, PPi, pyrophosphate, ROS, reactive oxygen species, TCA, trichloroacetic acid, ThrRS, threonyl-tRNA synthetase

## Abstract

Aminoacyl-tRNA synthetases (aaRSs) are enzymes that synthesize aminoacyl-tRNAs to facilitate translation of the genetic code. Quality control by aaRS proofreading and other mechanisms maintains translational accuracy, which promotes cellular viability. Systematic disruption of proofreading, as recently demonstrated for alanyl-tRNA synthetase (AlaRS), leads to dysregulation of the proteome and reduced viability. Recent studies showed that environmental challenges such as exposure to reactive oxygen species can also alter aaRS synthetic and proofreading functions, prompting us to investigate if oxidation might positively or negatively affect AlaRS activity. We found that while oxidation leads to modification of several residues in *Escherichia coli* AlaRS, unlike in other aaRSs, this does not affect proofreading activity against the noncognate substrates serine and glycine and only results in a 1.6-fold decrease in efficiency of cognate Ala-tRNA^Ala^ formation. Mass spectrometry analysis of oxidized AlaRS revealed that the critical proofreading residue in the editing site, Cys666, and three methionine residues (M217 in the active site, M658 in the editing site, and M785 in the C-Ala domain) were modified to cysteine sulfenic acid and methionine sulfoxide, respectively. Alanine scanning mutagenesis showed that none of the identified residues were solely responsible for the change in cognate tRNA^Ala^ aminoacylation observed under oxidative stress, suggesting that these residues may act as reactive oxygen species “sinks” to protect catalytically critical sites from oxidative damage. Combined, our results indicate that *E. coli* AlaRS proofreading is resistant to oxidative damage, providing an important mechanism of stress resistance that helps to maintain proteome integrity and cellular viability.

Aminoacyl-tRNA synthetases (aaRSs) are essential enzymes with a critical role in the translation of mRNA to produce proteins. AaRSs are involved in the attachment of the correct amino acid to its cognate tRNA (1). The process involves the activation of an amino acid in the active site of the aaRS in an ATP-dependent manner to form the intermediate aminoacyl-adenylate. The cognate tRNA is then recognized by the aaRS, and the activated amino acid is transferred to the 3′-CCA end of the tRNA. Finally, the aminoacyl-tRNA in complex with EF-Tu-GTP is transported to the ribosome to participate in translation. Many amino acids have similar physical and chemical properties making it challenging for some aaRSs to select the cognate from the amino acid pool in the cell. Although the active-site pocket of aaRSs can usually discriminate against the wrong amino acid, errors may still occur. To prevent these erroneous misactivated amino acids from being attached to tRNA or misaminoacylated tRNAs from participating in translation, half of the aaRSs in *Escherichia coli* have evolved proofreading mechanisms to maintain accurate translation (2). The aaRS proofreading mechanisms can either hydrolyze the misactivated amino acid in the active site (pretransfer editing) or hydrolyze the mischarged tRNA in specialized editing domains (post-transfer editing). Together, these proofreading mechanisms help maintain accurate protein synthesis, which facilitates cellular growth and adaptation (1, 3).

Cells across all domains of life are subject to varying environmental conditions that can directly impact proteins and their functions (4), and aaRS activity is frequently challenged by these stressors. The most common stress faced by a cell is oxidative stress since ATP production through the endogenous oxidative phosphorylation pathways results in the formation of reactive oxygen species (ROS) (5). Prolonged or acute exposure to ROS can induce both reversible and irreversible damage to DNA, RNA, proteins, and lipids (6, 7, 8, 9, 10). Proteins subjected to ROS may misfold and subsequently form protein aggregates (11). In addition, oxidation can affect a protein's enzymatic activity either directly (12), by changing a catalytic residue or indirectly (13), or by affecting its overall protein structure. Previous studies on the effects of aaRS oxidation have yielded the possibility of both modes of action. A critical cysteine residue in the editing site of threonyl-tRNA synthetase (ThrRS) was shown to be oxidized into a cysteine sulfenic acid (Cys-SOH) that impaired its proofreading activity. The oxidation of this single amino acid led to increased misincorporation of the noncognate serine at threonine codons (14, 15). In contrast, oxidation of phenylalanyl-tRNA synthetase (PheRS) caused an overall conformational change that led to elevated proofreading activity against its noncognate amino acid, *meta*-tyrosine (16, 17). It has also been observed that, upon oxidative stress, mammalian methionyl-tRNA synthetase (MetRS) can misaminoacylate several noncognate tRNAs leading to increased methionine incorporation in cellular proteins. The increase in methionine residues throughout the proteome is believed to act as a sink for oxygen radicals, thereby protecting the proteome from oxidative damage (18, 19, 20). These examples highlight the effect of oxidative stress on aaRSs and their contribution to translational regulation and fidelity.

Like ThrRS, alanyl-tRNA synthetase (AlaRS) encodes a distinct secondary editing domain, which hydrolyzes its noncognate substrates, glycine and serine, from tRNA^Ala^. The editing domains of AlaRS and ThrRS are homologous, even containing the same critical cysteine residue (21). Studies in higher eukaryotes have reported that increased mistranslation caused by a mutation in the AlaRS editing domain causes neurodegenerative disorders in mice (22, 23). Studies determining the threshold of mistranslation tolerated by *E. coli* showed that when AlaRS fidelity is reduced, there is global dysregulation of the proteome leading to slow growth, increased antibiotic sensitivity, and a decreased swimming motility (24). More recently, it has been observed that AlaRS editing defects in *Saccharomyces cerevisiae* leads to downregulation of carbon metabolism and the attenuation of protein synthesis and heat shock response (25). These findings indicate the high cost associated with defects in AlaRS fidelity, prompting us to investigate how oxidation of AlaRS impacts its editing activity.

We report that under oxidative stress, the conserved cysteine residue (C666) in the *E. coli* AlaRS editing site critical for proofreading is oxidized to a Cys-SOH, as is also the case in ThrRS; unlike the oxidation of *E. coli* ThrRS, this modification does not affect proofreading activity. Oxidation of *E. coli* AlaRS did result in a 1.6-fold decrease in cognate tRNA^Ala^ aminoacylation likely reducing the rate of formation of Ala-tRNA^Ala^. Therefore, by reducing the speed of tRNA^Ala^ aminoacylation and maintaining AlaRS proofreading under oxidative stress, the enzyme maintains translational fidelity by ensuring accurate Ala-tRNA^Ala^ formation. In addition, we observed that mutagenesis of the critical cysteine to alanine (C666A) in the editing site had a negative impact on the catalytic efficiency of cognate aminoacylation. These results indicate that the editing-site cysteine, C666, may play a role in cognate tRNA^Ala^ aminoacylation. Finally, mass spectrometry (MS) analyses revealed that every methionine residue in *E. coli* AlaRS is oxidized upon exposure to hydrogen peroxide (H_2_O_2_) indicating that the methionine residues may act as a sink for ROS, thereby protecting other critical amino acid residues from oxidative damage.

## Results

### *E. coli* AlaRS is subject to oxidation

It has been previously shown that enzymatic activities of *E. coli* ThrRS and *Salmonella typhimurium* PheRS are modulated under oxidative stress. To investigate whether oxidation affects AlaRS activity, recombinant AlaRS protein was purified from *E. coli* cells. An editing-deficient *E. coli* AlaRS protein harboring a mutation in the editing site (C666A) was also purified from *E. coli* cells. The editing-deficient AlaRS C666A protein is unable to hydrolyze misaminoacylated tRNA^Ala^ species and therefore accumulates Ser-tRNA^Ala^ and Gly-tRNA^Ala^ as determined by *in vitro* studies (21).

**Figure 1:**
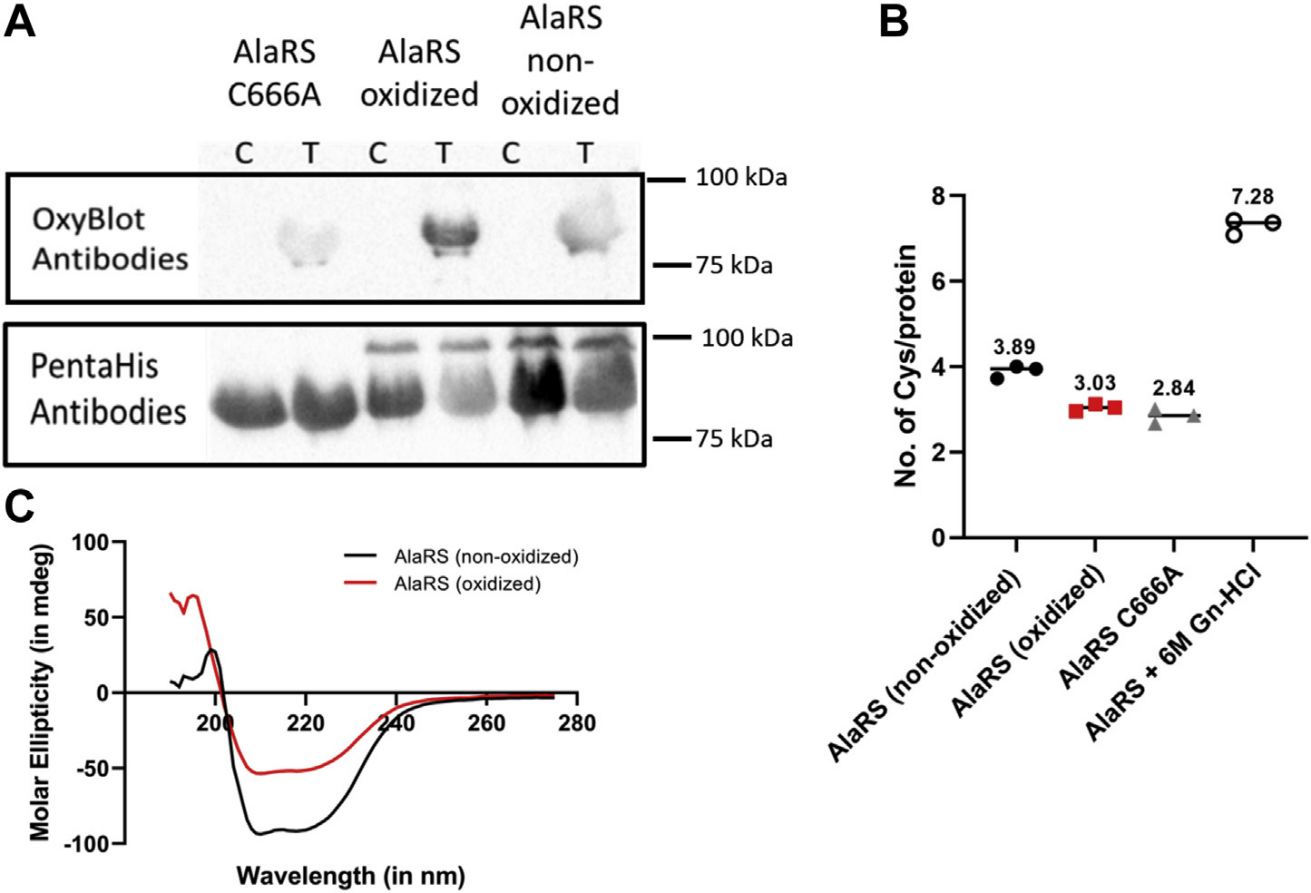
***Escherichia coli* AlaRS is subjected to protein oxidation.***A*, representative Western blot showing the oxidation of *E. coli* AlaRS. Lanes labeled T (test) have been derivatized using DNPH, whereas lanes labeled C (control) are not derivatized. Precision Plus Protein Kaleidoscope Prestained Protein Standards by Bio-Rad was used as the molecular weight marker and visualized by chemiluminescence imaging. *B*, quantification of the DTNB assay indicating the number of accessible cysteine residues per protein molecule. The error bars represent the standard deviation for three replicates. *C*, circular dichroism of nonoxidized *E. coli* AlaRS (*black*) and oxidized *E. coli* AlaRS (*red*) between 190 and 280 nm. AlaRS, alanyl-tRNA synthetase; DNPH, 2,4-dinitrophenylhydrazine; DTNB, thio-bis-(2-nitrobenzoic acid).

WT AlaRS protein was exposed to oxidative stress through treatment with 5 mM H_2_O_2_ *in vitro* for 1 h at 37 °C. The concentration of H_2_O_2_ used is likely higher than the physiological levels of ROS accumulation as *E. coli* cells grown in LB media in the presence of 5 mM H_2_O_2_ are unable to grow past the lag phase (data not shown). The oxidant was then dialyzed out to obtain the oxidized protein. Upon oxidation, carbonyl groups are introduced at lysine, arginine, proline, and threonine residues (26). These carbonyl groups were derivatized by the addition of 2,4-dinitrophenylhydrazine (DNPH) to form 2,4-dinitrophenylhydrazone. A reaction without the addition of DNPH was used as control. Immunoblotting analysis probing for the 2,4-dinitrophenylhydrazone residues showed that the AlaRS protein treated with H_2_O_2_ was subject to oxidation as the intensity of these bands was higher than their nonoxidized counterparts (Fig. 1*A*). As derivatization with DNPH will only reveal oxidative modifications at a few amino acid positions, further analyses using 5,5-dithio-bis-(2-nitrobenzoic acid) (DTNB) were performed to examine the extent of AlaRS oxidation. DTNB analysis allows for the quantification of free thiols in a protein and therefore is an indirect measure of the number of accessible cysteine residues in a protein (27). As cysteine oxidation was of particular interest based on previous ThrRS oxidation studies, DTNB analyses were performed on both oxidized and nonoxidized AlaRS. AlaRS treated with a denaturant, 6 M guanidine hydrochloride (Gn-HCl), and editing-deficient AlaRS C666A were used as controls. AlaRS treated with 6 M Gn-HCl was unfolded allowing the determination of seven accessible cysteine residues, whereas for the folded nonoxidized WT, four AlaRSs were detected. Consistent with the replacement of the cysteine in position 666 with alanine, editing-deficient AlaRS C666A reported three accessible cysteine residues. Surprisingly, DTNB analysis of the oxidized WT AlaRS protein indicated that three cysteine residues are accessible further suggesting that AlaRS is oxidized and that a cysteine residue may also be modified, rendering it inaccessible to react with DTNB (Fig. 1*B*). To ensure that oxidation did not affect the overall conformation of the protein as observed with *S. typhimurium* PheRS oxidation, the secondary structure of nonoxidized and oxidized *E. coli* AlaRS was assessed using circular dichroism (Fig. 1*C*). Circular dichroism is a method that determines the secondary structure of the protein based on the shape of the molar ellipticity curve. Despite differences in the magnitude of their signals, both the nonoxidized and oxidized AlaRS samples shared similar profiles across the wavelengths that were monitored suggesting that there is no significant change in the secondary structure of the protein upon oxidation. The difference in the curves may be because of a slight loss of α-helices upon oxidation. However, changes in the spectra are not suggestive of global modification to the protein's secondary structure, and further biophysical and structural studies are required to understand the function of *E. coli* AlaRS under oxidative stress. Combined, these results indicate that oxidation of *E. coli* AlaRS results in the chemical modification of amino acid residues without significantly affecting the overall conformation of the protein.

### Identification and mapping of *E. coli* AlaRS residues affected by oxidation

Amino acids are highly susceptible to attack by ROS produced during oxidative stress (28). Among the 20 proteinogenic amino acids, aromatic amino acids and sulphur-containing amino acids are the most sensitive to chemical modification through oxidation (29). Cysteine and methionine are the two sulphur-containing amino acids that upon oxidation are degraded to yield Cys-SOHs and methionine sulfoxides (Met-SOs), respectively (26). Previous studies on the effect of oxidation on *E. coli* ThrRS revealed oxidative modification of the cysteine residue, C182, in the proofreading domain, results in reduced proofreading of ThrRS against its noncognate substrates (14).

**Figure 2:**
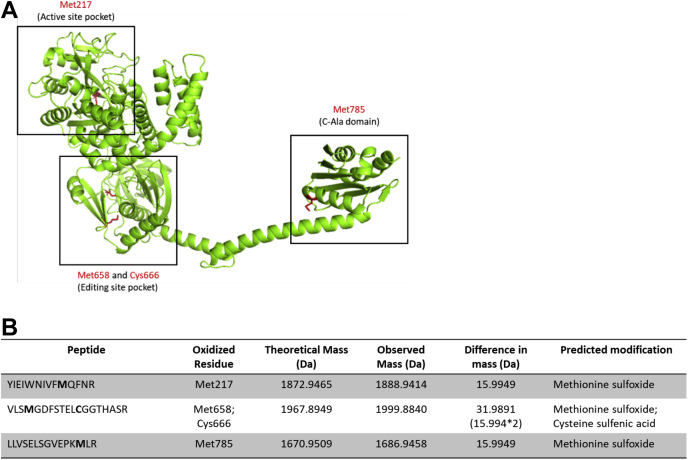
**Identification and mapping of AlaRS residues oxidized upon treatment with H**_**2**_**O**_**2**_**.***A*, the residues Met217, Met658, Cys666, and Met785 were identified by mass spectrometry and mapped to the different domains using the *Escherichia coli* AlaRS alpha-fold predicted structure. *B*, the mass difference between the nonoxidized and oxidized residues/peptides was calculated to identify the oxidative modification. The methionine residues (Met217, Met658, and Met785) were oxidized to a methionine sulfoxide, whereas the cysteine residue (Cys666) was oxidized to a cysteine sulfenic acid, when treated with H_2_O_2_. AlaRS, alanyl-tRNA synthetase; H_2_O_2_, hydrogen peroxide.

To obtain the site-specific landscape of AlaRS oxidation, MS was performed to identify all the cysteine and methionine residues in the protein that were susceptible to oxidation (Fig. S1). Purified recombinant *E. coli* AlaRS protein and oxidized *E. coli* AlaRS protein treated with H_2_O_2_ were separated on a denaturing polyacrylamide gel. The bands containing the proteins were excised, treated with trypsin to fragment the protein into peptides, and analyzed by LC–MS/MS (30). The results were filtered for 1% false discovery rate at the peptide level. The data were first analyzed using Proteome Discoverer against a database that contained *E. coli* AlaRS sequence to qualitatively identify oxidized cysteine and methionine residues. Our analysis revealed that three methionine residues, Met217, Met658, and Met785, and one cysteine residue, Cys666, the critical cysteine homologous to Cys182 in ThrRS, were modified in the oxidized protein (Fig. S2). Using the AlphaFold modeling predictions (31), the identified residues were mapped onto the *E. coli* AlaRS structure. Based on these predictions, Met217 is in the active-site pocket (32, 33), Met658 and Cys666 are positioned in the editing domain (21), and Met785 is in the C-Ala domain (34, 35) (Fig. 2*A*).

To identify the oxidation products formed when the cysteine and methionine residues are treated with H_2_O_2_, the difference between the theoretical mass of the peptide and the observed mass of the peptide was calculated using PEPTIDEMASS, an online tool by ExPasy (36). The MS data indicate that difference between the theoretical and observed mass of the Cys666 residue upon oxidation was 15.9949 Da, which corresponds to the mass of one oxygen atom. Therefore, under oxidative stress, the Cys666 residue is modified to a Cys-SOH (37) (Fig. 2*B*). Similarly, the differences between the theoretical and observed masses of the Met217, Met658, and Met785 residues upon oxidation were 15.9949 Da. Thus, the methionine residues undergo oxidative modification by the addition of one oxygen atom forming Met-SO (38) (Fig. 2*B*). The MS data revealed that every methionine residue of *E. coli* AlaRS was oxidized when exposed to H_2_O_2_, indicating that the methionine residues may act as a sink for ROS, thereby protecting other critical amino acid residues from oxidative damage.

### Oxidation of *E. coli* AlaRS reduces the rate of cognate tRNA^Ala^ aminoacylation

Oxidative modifications of amino acid residues have previously been shown to regulate the function of several enzymes. To understand how oxidative stress regulates the canonical aminoacylation activity of AlaRS, *in vitro* amino acid activation and aminoacylation kinetic assays were performed using oxidized and nonoxidized AlaRS.

The first step of the AlaRS aminoacylation reaction is the activation of alanine in the presence of ATP to form the aminoacyl-adenylate (1). To determine if oxidation leads to any change in alanine activation, pyrophosphate (PPi) exchange was conducted and the steady-state activation kinetics were determined. The results of this experiment determined that oxidation causes a 1.5-fold increase in the catalytic efficiency (*k*_cat_/*K*_*M*_) of alanine activation (Table 1). The second step of the aminoacylation reaction is the transfer of the activated aminoacyl-adenylate onto the tRNA (1). To determine if AlaRS oxidation affects Ala-tRNA^Ala^ formation, *in vitro* steady-state aminoacylation kinetics were performed. The results of this experiment identified that oxidation results in a 1.6-fold decrease in the catalytic efficiency for tRNA^Ala^ aminoacylation (Table 1). Combined, these results indicate that although oxidation of *E. coli* AlaRS increases the rate of alanine activation, there is still a moderate decrease in tRNA^Ala^ turnover and tRNA^Ala^ affinity, which leads to a reduction in cognate tRNA^Ala^ aminoacylation rate.

**Table 1:** Steady-state kinetics of *E. coli* AlaRS under oxidative stress

H_2_O_2_ treated	Alanine				tRNA^Ala^			
	*k*_cat_ (min^−1^)	*K*_*M*_ (μM)	*k*_cat_/*K*_*M*_ (μM^−1^ min^−1^)	Relative catalytic efficiency	*k*_cat_ (min^−1^)	*K*_*M*_ (μM)	*k*_cat_/*K*_*M*_ (μM^−1^ min^−1^)	Relative catalytic efficiency
No	962 ± 243	329 ± 84.6	2.9	1	9.9 ± 0.5	1.1 ± 0.1	9.0	1
Yes	1600 ± 298	355 ± 90.4	4.5	1.5	3.7 ± 0.2	0.7 ± 0.2	5.5	0.6

The errors represent the standard deviation for three replicates.

### *E. coli* AlaRS proofreading is not affected by oxidative stress

Previous studies exploring the effect of oxidative stress on *E. coli* ThrRS determined that oxidation of a critical cysteine residue (Cys182) in the editing site of ThrRS is sufficient to cause a significant decrease in the rate of hydrolysis of the misaminoacylated Ser-tRNA^Thr^ species (14). It has been shown that *E. coli* ThrRS and *E. coli* AlaRS share the same editing domain architectures including a conserved critical cysteine residue (21). Our MS analysis revealed that the Cys666 residue in the editing site of *E. coli* AlaRS is oxidized, as is the homologous critical cysteine in the *E. coli* ThrRS editing site. To determine if oxidation causes any change in the editing activity of AlaRS as observed in ThrRS, *in vitro* proofreading assays were performed. Purified recombinant *E. coli* AlaRS protein and *in vitro* transcribed *E. coli* tRNA^Ala^ were used in all experiments. Oxidized *E. coli* AlaRS treated with H_2_O_2_ was prepared as previously described. The editing-deficient AlaRS C666A protein that is unable to hydrolyze Ser-tRNA^Ala^/Gly-tRNA^Ala^ was used as a control.

**Figure 3:**
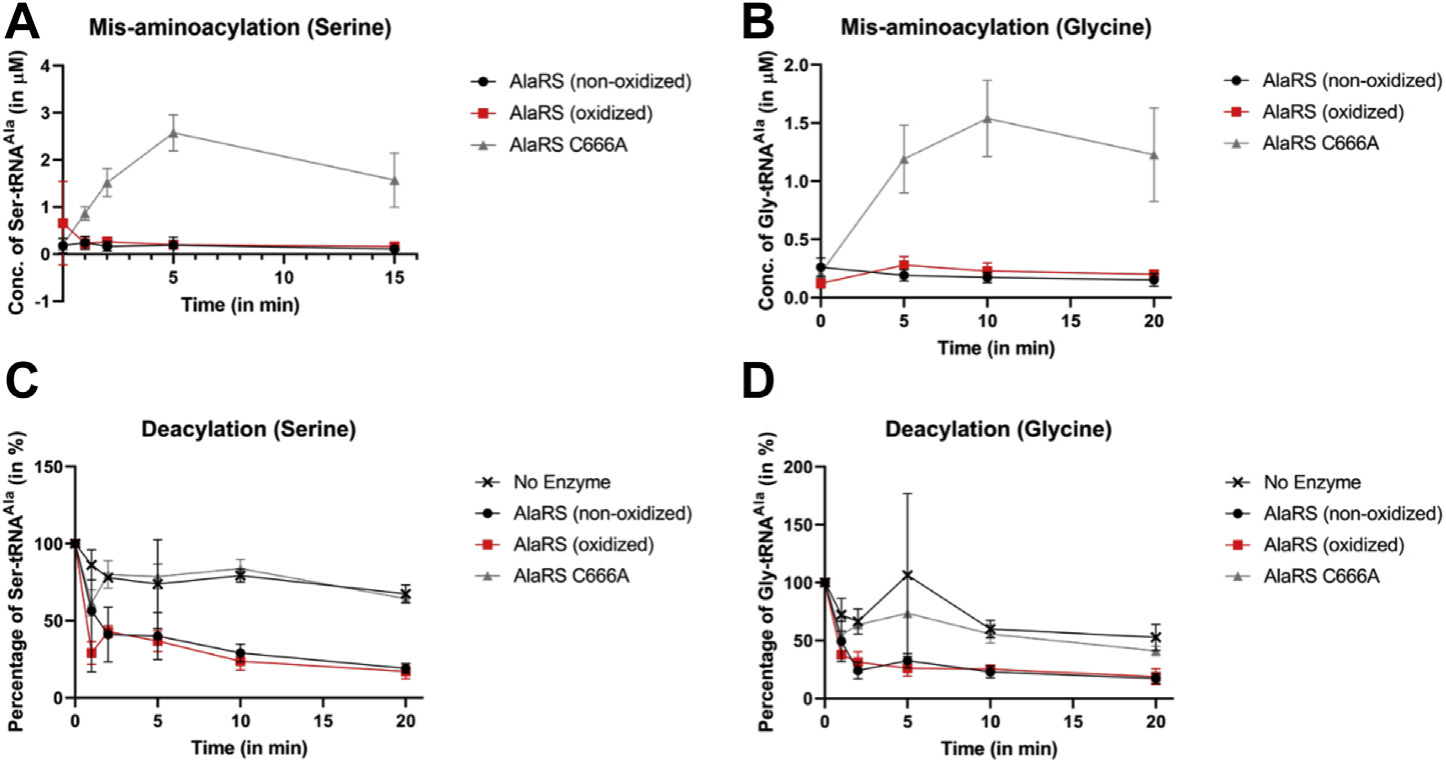
**Oxidation does not alter proofreading activity of AlaRS.** Misaminoacylation assays using the noncognate amino acids, (*A*) serine and (*B*) glycine to monitor proofreading activity of AlaRS under oxidative stress. Deacylation assays showing the hydrolysis of (*C*) Ser-tRNA^Ala^ and (*D*) Gly-tRNA^Ala^ to monitor proofreading activity of AlaRS upon oxidation. Editing-deficient AlaRS C666A was used as a control in all the experiments. The error bars represent the standard deviation for three replicates. AlaRS, alanyl-tRNA synthetase.

Misaminoacylation assays were performed to determine changes in the formation of noncognate Ser-tRNA^Ala^/Gly-tRNA^Ala^ species upon oxidation. If the enzyme maintains its proofreading activity, there will be no accumulation of misaminoacylated tRNA^Ala^ as the enzyme would successfully hydrolyze the noncognate substrate. If the proofreading activity was altered by oxidation, there would be accumulation of misaminoacylated tRNA^Ala^. While the editing-deficient AlaRS C666A accumulated Ser-tRNA^Ala^ and Gly-tRNA^Ala^ over time, both the nonoxidized and oxidized AlaRS were unable to form the misaminoacylated tRNA^Ala^ species indicating that oxidation by H_2_O_2_ does not affect the editing activity of AlaRS (Fig. 3, *A* and *B*).

To further validate this observation, AlaRS editing in *trans* was analyzed using deacylation assays. Preformed Ser-tRNA^Ala^/Gly-tRNA^Ala^ was incubated with the different AlaRS enzymes to directly assess hydrolytic activity over time. While the editing-deficient AlaRS C666A was unable to deacylate either Ser-tRNA^Ala^ or Gly-tRNA^Ala^, the nonoxidized and oxidized AlaRS enzymes exhibited similar levels of Ser-tRNA^Ala^ and Gly-tRNA^Ala^ deacylation. Combined, these results indicate that oxidation of *E. coli* AlaRS does not affect its noncognate substrate proofreading activity (Fig. 3, *C* and *D*).

### *In vitro* characterization of the AlaRS oxidation variants

To assess the roles of the oxidized cysteine and methionine residues identified by MS, mutant proteins were cloned using site-directed mutagenesis. The identified oxidized residues were mutated to encode alanine codons to generate oxidation-ablative mutants. Alanine was chosen because it is known to cause the least physical disruption to the secondary structure of the protein and alanine is not subject to oxidation (39). The plasmid containing the *E. coli* His-tagged AlaRS was used as the template for mutagenesis.

**Figure 4:**
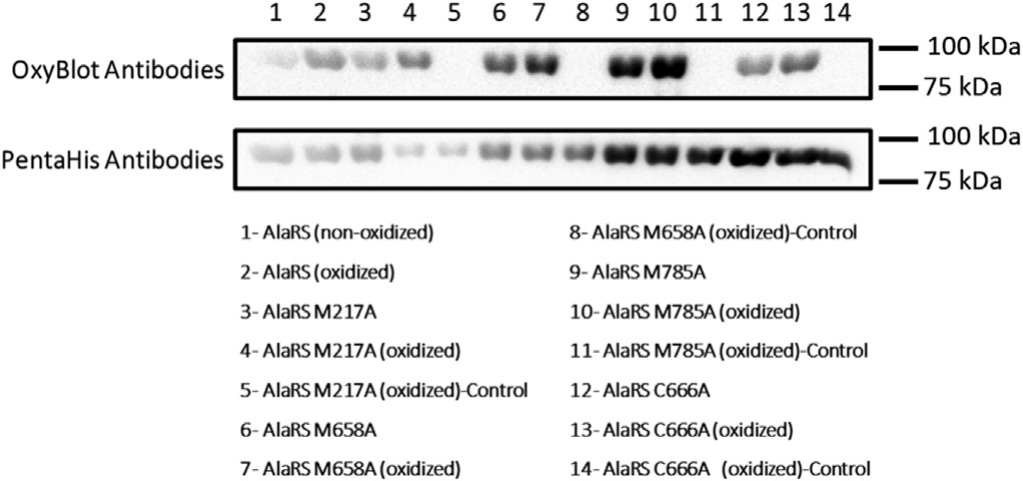
***Escherichia coli* AlaRS variants are subject to oxidation.** Representative Western blot showing the oxidation of the AlaRS oxidation variants. The lanes marked as control are not derivatized and hence do not interact with the antibody. Precision Plus Protein Kaleidoscope Prestained Protein Standards by Bio-Rad was used as the molecular weight marker and visualized by chemiluminescence imaging. AlaRS, alanyl-tRNA synthetase.

**Figure 5:**
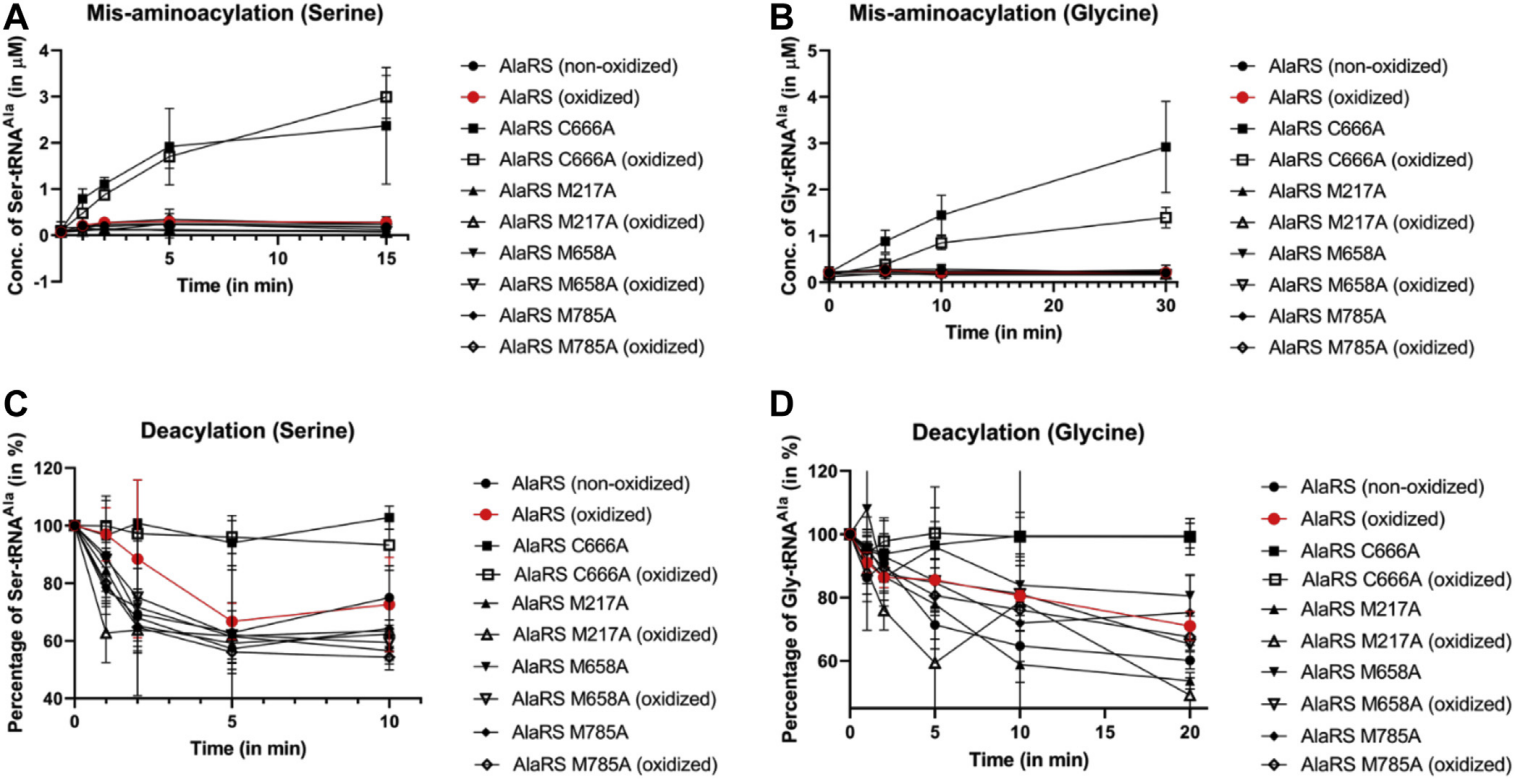
**Oxidative modification of the identified residues does not alter the proofreading activity of *Escherichia coli* AlaRS.** Misaminoacylation assays were conducted using the noncognate amino acids, (*A*) serine and (*B*) glycine to monitor proofreading activity of the AlaRS oxidation variants. Deacylation assays showing the hydrolysis of (*C*) Ser-tRNA^Ala^ and (*D*) Gly-tRNA^Ala^ were done to monitor proofreading activity of the AlaRS oxidation variants. The errors represent the standard deviation for three replicates. AlaRS, alanyl-tRNA synthetase.

To confirm that the protein variants (M217A, M658A, C666A, and M785A) were subject to oxidation, after H_2_O_2_ treatment, the proteins were derivatized by the addition of DNPH followed by immunoblotting analysis as described previously. The results indicated that all the AlaRS oxidation variants were oxidized as shown by the higher intensity bands when treated with H_2_O_2_ (Fig. 4). Since oxidation of the WT *E. coli* AlaRS protein did not affect the proofreading activity, we wanted to confirm that all the AlaRS oxidation variants retained proofreading activity as well. *In vitro* misaminoacylation and deacylation assays were performed as described previously. The results of these experiments suggested that while mutagenesis of the critical cysteine, C666, in the editing site into alanine reduces AlaRS proofreading, oxidative modification of amino acid residues does not impact AlaRS proofreading activity (Fig. 5).

Previous steady-state aminoacylation kinetics performed with oxidized *E. coli* AlaRS indicated a 1.6-fold reduction in cognate tRNA^Ala^ aminoacylation (Table 1). To assess the contribution of each residue to the regulation of AlaRS under oxidative stress, *in vitro* steady-state aminoacylation kinetic assays were performed with the AlaRS variants (Table 2). Observations from this experiment show that alanine replacement of the critical cysteine (C666) in the editing site results in a 7.5-fold decease in the catalytic efficiency as compared with the WT AlaRS enzyme. This indicates that the critical cysteine, C666, known to be involved in the discrimination against noncognate Ser/Gly-tRNA^Ala^ in the editing domain active site also interacts with the AlaRS active-site pocket affecting tRNA^Ala^ aminoacylation. However, the catalytic efficiencies of the AlaRS variants (WT, M217A, M658A, C666A, and M785A) upon oxidation with H_2_O_2_ decreases uniformly as compared with their nonoxidized counterparts. Combined, these results indicate that while Cys666 may play a role in cognate tRNA^Ala^ aminoacylation, none of the methionine residues identified are solely responsible for the decrease in AlaRS catalytic efficiency observed under oxidative stress.

**Table 2:** Steady-state aminoacylation kinetics of the AlaRS oxidation variants using tRNA^Ala^

Enzyme	*K*_*M*_ (μM)	*k*_cat_ (min^−1^)	*K*_*M*_/*k*_cat_ (min^−1^ μM^−1^)	Relative catalytic efficiency
AlaRS (nonoxidized)	1.1 ± 0.1	9.9 ± 0.5	9.0	1.0
AlaRS (oxidized)	0.7 ± 0.2	3.7 ± 0.2	5.5	0.6
AlaRS M217A	0.9 ± 0.1	6.9 ± 0.1	7.8	0.9
AlaRS M217A (oxidized)	1.3 ± 0.1	6.7 ± 0.4	5.1	0.6
AlaRS M658A	1.7 ± 0.3	11.5 ± 0.7	6.6	0.7
AlaRS M658A (oxidized)	0.7 ± 0.1	4.4 ± 0.2	6.0	0.7
AlaRS M785A	1.2 ± 0.2	5.5 ± 0.3	4.8	0.5
AlaRS M785A (oxidized)	1.9 ± 0.7	5.4 ± 0.7	2.9	0.3
AlaRS C666A	1.6 ± 0.8	2.3 ± 0.4	1.5	0.2
AlaRS C666A (oxidized)	2.0 ± 0.4	2.3 ± 0.1	1.1	0.1

The errors represent the standard deviation for three replicates.

## Discussion

### *E. coli* AlaRS proofreading activity is protected against oxidative stress

During normal growth and development, cells are subjected to oxidative stress, leading to changes in the structure and function of cellular proteins. As aaRSs are required to maintain the pool of aminoacyl-tRNA used by the ribosome for protein production, it is vital to regulate aaRSs activity during oxidative stress to prevent changes in the rate and fidelity of protein translation (40, 41). Previous studies on the effect of oxidative stress on ThrRS revealed that exposure to H_2_O_2_ oxidizes a critical cysteine residue (C182) in the editing site of ThrRS leading to the accumulation of Ser-tRNA^Thr^ (14). While the DTNB assay showed four accessible cysteine residues, our MS data revealed the oxidation of one cysteine, Cys666, in the editing site of AlaRS. Studies on the mechanism of oxidation in *Ec* ThrRS have shown that all cysteine residues in a protein are not equally susceptible to oxidation (15). The susceptibility of cysteine residues to oxidation depends on the protein microenvironment, including type of oxidant used, solvent accessibility, p*K*a, and polarity of neighboring residues. Cysteine sulfenylation requires deprotonated cysteine residues, as the nucleophilic thiolate (RS^−^) can attack the oxidant, H_2_O_2_ (42). In the *Ec* ThrRS structure, adjacent histidine residues stabilize the thiolate form making the editing-site cysteine, C182, susceptible to oxidative modification (15). Since *Ec* ThrRS and *Ec* AlaRS have homologous editing domains, the deprotonated editing site, cysteine, Cys666, is likely stabilized in a similar way leading to the formation of a Cys-SOH upon oxidation with H_2_O_2_. However, unlike ThrRS oxidation, modification of C666 of AlaRS did not result in a proofreading defect. Instead, the oxidation of AlaRS has a modest negative impact on cognate aminoacylation likely reducing the speed of Ala-tRNA^Ala^ synthesis *in vivo*.

In *E. coli*, mutagenesis of the C666 residue in the AlaRS-editing domain results in numerous phenotypic defects, such as reduced swimming motility, increased antibiotic sensitivity, and reduced heat stress response. The same study also revealed that mutagenesis of the C182 residue in the ThrRS editing domain does not elicit the same detrimental response, indicating that under certain growth conditions, AlaRS proofreading is more important for cell viability, in turn suggesting that Ala-to-Ser mistranslation is potentially more problematic than Thr-to-Ser decoding errors (24). This earlier finding is consistent with our results reported here that AlaRS proofreading is protected against destructive oxidation because of the high cost of AlaRS-mediated mistranslation. Our findings further indicate that any cost to the cell arising from a reduction in the rate of tRNA^Ala^ aminoacylation under oxidative stress is more than offset by the benefits of maintaining AlaRS proofreading and ensuring accurate Ala-tRNA^Ala^ formation.

### Contribution of the editing-site cysteine (C666) in tRNA^Ala^ aminoacylation

The editing site of *E. coli* AlaRS is responsible for discriminating between the cognate alanine and noncognate serine and glycine residues. The editing site of AlaRS contains conserved HXXH and CXXXH motifs thought to constitute a zinc-binding motif (43). The conserved histidine and cysteine (C666) residues coordinate a zinc ion, which in turn recognizes the β-hydroxyl group of serine, thereby aiding in discrimination (44). Once the noncognate amino acids are recognized, they can be hydrolyzed from the 3′-end of the tRNA in the editing site, to prevent serine from being misincorporated at alanine codons. Additional studies have reported that the conserved cysteine, C666, in the editing site participates in multiple interactions that stabilize aminoacyl-tRNA binding (43). While the C666 residue plays a critical role in discrimination against serine in the editing site, our results indicate that loss of C666 may also affect cognate aminoacylation in the active site. Mutagenesis of the cysteine residue to alanine (C666A) not only results in reduced proofreading against Ser-tRNA^Ala^ and Gly-tRNA^Ala^ but also greatly reduces cognate aminoacylation likely resulting in reduced Ala-tRNA^Ala^ formation. Although, the active sites and editing sites of aaRSs can recognize tRNA^Ala^ independently, previous studies have concluded that tRNA^Ala^ recognition by both domains is necessary for the translocation of the aminoacyl-tRNA into the editing site (45). However, our results indicate that C666 may interact and stabilize the tRNA^Ala^ prior to translocation. Molecular dynamic simulation studies looking into the editing domain (INS domain) of prolyl-tRNA synthetase identified contiguous pathways of residue–residue interactions between the aminoacylation and editing domains of *E. coli* prolyl-tRNA synthetase that maintain dynamic coupling between the two domains (46, 47). In addition, the study showed that deletion of the INS domain or mutagenesis of conserved residues in the INS domain disrupts the internal protein dynamics leading to reduced cognate aminoacylation (48). Thus, while oxidative modification of the C666 residue in the editing site does not affect proofreading, it may destabilize the tRNA^Ala^ interactions and residue–residue interactions between the aminoacylation and editing domains of AlaRS resulting in reduced catalytic efficiency during aminoacylation.

### Oxidation of AlaRS methionine residues likely protects AlaRS from oxidative damage

Previous studies have found that, under oxidative stress, exposure to ROS results in the phosphorylation of MetRS (18). This phosphorylated MetRS has an increased affinity for noncognate tRNA species leading to higher rates of tRNA mismethionylation. Ultimately, this increased tRNA methionylation leads to a higher incidence of methionine residues being incorporated into cellular proteins (18). Methionine residues are highly susceptible to oxidation by ROS because of their sulphur-containing side chains (26) and therefore act as a sink for ROS, reducing their levels in the cell. This inaccuracy of MetRS therefore seems to be a defense mechanism against oxidative stress. The MS analysis of oxidized *E. coli* AlaRS revealed that that every methionine residue of *E. coli* AlaRS was oxidatively modified when exposed to H_2_O_2_. A multiple sequence alignment of the AlaRS proteins from several prokaryotic and eukaryotic model systems indicate that the methionine residues found to be oxidized upon H_2_O_2_ treatment are not evolutionarily conserved. Furthermore, alanine scanning of the methionine residues indicates that these residues do not have any structural or functional roles that impact aminoacylation, supporting the notion that they act as ROS sinks. Thus, the modification of the methionine residues of *E. coli* AlaRS likely protects other critical amino acid residues from oxidative damage.

Work from our group and others has shown that the proofreading activities of aaRSs, like PheRS and ThrRS, can be modulated by oxidative stress. Through this work, we have identified that although oxidative stress can modify the critical residues of aaRSs in similar ways, the effect of this modification on the aaRSs enzymatic activity is varied. Oxidation of the editing-site cysteine to Cys-SOH in *E. coli* ThrRS reduces proofreading, whereas the same modification in *E. coli* AlaRS maintains the proofreading activity. Furthermore, we identified that alanine replacement of the critical cysteine (Cys666) leads to a defect in tRNA^Ala^ aminoacylation. This suggests that the global dysregulation of the *E. coli* proteome observed upon the perturbation of AlaRS editing is a result of both Ala-to-Ser misincorporation and reduction of tRNA^Ala^ aminoacylation. We also identified three methionine residues (Met217 in the active site, Met658 in the editing site, and Met785 in the C-Ala domain) that are oxidized to Met-SO. Alignment of the *E. coli* AlaRS protein with the sequences from 12 model organisms shows that the methionine residues identified are not evolutionarily conserved and do not have any structural or functional roles that impact aminoacylation. Thus, the oxidation of these methionine residues may act as ROS “sinks” to protect catalytically critical sites from oxidative damage. While oxidative stress may influence the levels of mistranslation, as seen with ThrRS and PheRS, *E. coli* AlaRS activity is maintained to prevent proteome dysregulation.

## Experimental procedures

### *E. coli* AlaRS purification and construction of AlaRS oxidation variants

The *E. coli alaS* gene was cloned into the pET21b expression vector with a C-terminal His tag and purified from *E. coli* BL21(DE3) cells. Point mutations encoding amino acid substitutions (M217A, M658A, and M785A) were introduced into the *alaS* gene through PCR site-directed mutagenesis using two self-complementary oligonucleotides carrying the appropriate mutations (49). Following PCR mutagenesis, the reaction was treated with DpnI for 4 h at 37 °C to digest the template plasmid. The plasmids were then sequenced to confirm the introduction of the desired mutations.

The production of the AlaRS variants was induced at 37 °C for 4 h with 1 mM IPTG in LB media. The cells were spun down and resuspended in a buffer containing 50 mM Tris–HCl (pH 8.0), 300 mM NaCl, 10 mM imidazole, and EDTA-free cOmplete Mini Protease Inhibitor (Sigma). The cells were then lysed by sonication, clarified, and purified using a TALON metal affinity resin. AlaRS was eluted using 250 mM imidazole. The fractions containing the protein were concentrated using a 10 K Amicon concentrator and dialyzed into a final buffer containing 50 mM Tris–HCl (pH 7.5), 300 mM NaCl, and 50% glycerol (50).

### Oxidation of AlaRS

The purified protein was oxidized with 5 mM H_2_O_2_ at 37 °C for 15 min. The excess H_2_O_2_ was removed using a 10 K Amicon concentrator. The concentrations of the nonoxidized and oxidized proteins were determined by spectrophotometric analysis at 280 nm (16). Successful protein oxidation was confirmed using the Millipore OxyBlot Protein Oxidation Detection Kit. About 20 μg of protein was combined with 12% SDS and either DNPH or the control solution and incubated at room temperature for 30 min. The reaction was quenched using the proprietary neutralization buffer. The derivatized proteins were then run on a 10% SDS-PAGE gel and transferred onto a nitrocellulose membrane. Western blot analysis was performed with antibody dilutions per the manufacturer’s recommendations and analyzed using ImageJ (NIH) (51). The blot was then stripped off the anti-DNPH antibodies by incubating the nitrocellulose membrane in a stripping buffer (0.75 g glycine, 0.05 g SDS, 0.5 ml Tween-20 in 50 ml water, pH 2.2) for 10 min. This was followed by three washes in 1× Tris-buffered saline and three washes in 1× Tris-buffered saline with Tween-20 for 5 min each. The membrane was subsequently blocked with a solution of skim milk and blotted with 1° antibody Penta-His (1:2000 dilution) and 2° antibody antimouse horseradish peroxidase (1:5000 dilution). Precision Plus Protein Kaleidoscope Prestained Protein Standards by BioRad was used as the molecular weight marker and visualized by chemiluminescence imaging. The blot was then imaged and analyzed in ImageJ (51). The extent of protein oxidation was quantified by normalization to the Penta-his blot (loading control).

### DTNB assay

To measure cysteine oxidation, a DTNB assay was carried out in a 96-well plate. Equal volume of Ellman's reagent/DTNB was added to protein aliquots (nonoxidized *E. coli* AlaRS, oxidized *E. coli* AlaRS, *E. coli* AlaRS C666A, and *E. coli* AlaRS + 6 M Gn-HCl) stored in the reaction buffer containing sodium phosphate salt and 0.5 M EDTA. The reaction was allowed to proceed at room temperature for 15 min. The absorbance at 412 nm was recorded using a plate reader. A standard curve was obtained using DTNB with increasing concentrations of l-cysteine (0–1.5 mM). The absorbance readings obtained after treating the AlaRS variants with DTNB were then fitted on to the standard curve to assess the number of accessible cysteines.

### Circular dichroism

Purified AlaRS proteins were oxidized as described previously. Buffer exchange was then performed using a 0.5 ml Amicon Ultra Centrifugal filter tube to resuspend the oxidized and nonoxidized AlaRS in 100 mM KH_2_PO_4_. Purified proteins (0.5 mg/ml) were analyzed in a quartz absorption cuvette with a 1 mm path length using a Jasco J-815 Circular Dichroism Spectrometer. Molar ellipticity (θ) values between 190 and 280 nm were recorded.

### PPi exchange

PPi exchange assays were performed at 37 °C in HKM buffer (100 mM Na-Hepes, pH 7.2, 30 mM KCl, 10 mM MgCl_2_, 2 mM NaF) containing 2 mM ATP, 2 mM (^32^P) PPi (2–4 cpm/pmol), and 10 nM of the purified enzyme. The concentrations of the substrate alanine ranged from 0 to 1 mM. The reactions were quenched at the selected time points by aliquoting 25 μl of the reaction into 970 μl of a charcoal solution (1% charcoal, 5.6% HClO_4_, and 75 mM PPi). The quenched solution was then spotted onto 3 MM Whatman filter discs, vacuum filtered, and washed three times with 5 ml water. The discs were then dried, and the radioactivity was determined by liquid scintillation counting. Results were fitted using Prism, a software by GraphPad Inc to a Michaelis–Menten curve to determine the amino acid activation kinetics (52).

### *In vitro* transcription of tRNA^Ala^

The most abundant *E. coli* tRNA^Ala^ isoacceptor gene (*alaT*/*alaU*/*alaV*) was cloned into a pUC18 plasmid with a T7 promoter sequence. The pUC18 plasmid containing the *E. coli* tRNA^Ala^ gene was used as a template for PCR amplification. The PCR product was purified and used as the template for *in vitro* transcription to make tRNA^Ala^. The *in vitro* transcription reaction was performed using the PCR-amplified product and T7 RNA polymerase suspended in a buffer containing 40 mM Tris, pH 8.0, 2 mM spermidine, 22 mM MgCl_2_, 5 mM DTT, 50 μg/ml bovine serum albumin, 0.2 M 5′GMP, pyrophosphatase, 4 mM ATP, 4 mM GTP, 4 mM CTP, 4 mM TTP, and RNase inhibitor. The reaction was incubated at 42 °C for 12 to 16 h. The tRNA^Ala^ was then purified with diethylaminoethyl cellulose resin and eluted using a 1 M NaCl. The fractions containing tRNA were pooled and precipitated using 1/10th volume of 3 M sodium acetate (pH 5.5) and 3× volume of ethanol at −80 °C overnight. The precipitated tRNA was pelleted at 5000*g* for 25 min, dried, and resuspended in RNase-free water (50). The concentration of active tRNA was determined by aminoacylation reactions.

### Steady-state aminoacylation kinetics

Enzyme activity was monitored by steady-state aminoacylation assays performed with both the nonoxidized and oxidized AlaRS. To a reaction mixture containing 50 nM AlaRS (nonoxidized/oxidized), 0 to 30 μM tRNA^Ala^ and 8 mM ATP in appropriate buffers (0.5 M Hepes [pH 7.2], 150 mM KCl, and 50 mM MgCl_2_), 50 μM of [^14^C]-alanine was added, and incubated at 37 °C. At minute intervals (0–4 min), a fixed volume of the reaction mixture was spotted onto membranes presoaked with 5% trichloroacetic acid (TCA). The membranes were then washed three times with 5% TCA followed by one wash with 70% ethanol. The membranes were then dried, and the radioactivity was quantified by liquid scintillation counting. Results were fitted to a Michaelis–Menten curve using GraphPad Prism to determine the steady-state activation kinetics (52).

### Proofreading assays

To monitor changes in the proofreading activity between the AlaRS variants, misaminoacylation and deacylation assays were performed.

#### Misaminoacylation assays

To a reaction mixture containing 5 μM AlaRS (nonoxidized/oxidized), 5 μM tRNA^Ala^ and 8 mM ATP in appropriate buffers (0.5 M Hepes [pH 7.2], 150 mM KCl, and 50 mM MgCl_2_), 750 μM [^14^C]-glycine/750 μM [^3^H]-serine was added and incubated at 37 °C. At regular intervals (0–30 min), a fixed volume of the reaction mixture was spotted onto a presoaked 5% TCA membrane. The radiolabeled aminoacyl-tRNA was quantified by a liquid scintillation counting.

#### Deacylation assays

To generate preformed aminoacyl-tRNA^Ala^, *E. coli* EF-Tu was activated (EF-Tu expression construct was provided by Dr Kurt Fredrick, The Ohio State University). About 50 μM of EF-Tu was added to 50 mM Tris (pH 7.8), 100 μM DTT, 68 mM KCl, 6.7 mM MgCl_2_, 2.5 mM phosphoenol pyruvate, 30 μg pyruvate kinase, and 500 μM GTP and incubated at 37 °C for 30 min. The activated EF-Tu was used immediately for misaminoacylation reactions. Preformed Gly-tRNA^Ala^/Ser-tRNA^Ala^ was generated by adding 10 μM tRNA^Ala^ to 950 μM (^14^C)-Gly/950 μM (^3^H)-Ser, 5 μM activated EF-Tu, 5 μM *E. coli* AlaRS C666A, 8 mM ATP, and aminoacylation buffer (0.5 M Hepes [pH 7.2], 150 mM KCl, and 50 mM MgCl_2_) and incubated at 37 °C for 1 h. The reaction was quenched by adding an equal volume of acid phenol–chloroform. The top aqueous layer containing Gly-tRNA^Ala^/Ser-tRNA^Ala^ was separated and precipitated using 1/10th volume of 3 M sodium acetate (pH 5.5) and 3× volume of ethanol at −80 °C overnight. The precipitated tRNA was pelleted at 16,800*g* for 25 min, dried, and resuspended in 100 mM sodium acetate (pH 4.5). To a reaction mixture containing the Gly-tRNA^Ala^/Ser-tRNA^Ala^ in appropriate buffers (0.5 M Hepes [pH 7.2], 150 mM KCl, and 50 mM MgCl_2_), 150 nM AlaRS (nonoxidized/oxidized) was added and incubated at 37 °C. At regular intervals (0–20 min), a fixed volume of the reaction mixture was spotted onto a presoaked 5% TCA membrane. The radiolabeled aminoacyl-tRNA was quantified by liquid scintillation counting.

### MS

Purified AlaRS protein was oxidized with 5 mM H_2_O_2_ at 37 °C for 15 min. The excess H_2_O_2_ was removed using a 10 K Amicon concentrator. The samples were run on a 12% SDS-PAGE gel. The protein samples were extracted from the gel and digested using trypsin for LC–MS/MS analysis (Fred Hutchinson Cancer Research Center). The peptides were then analyzed using Proteome Discoverer to identify the oxidized residues. The difference between the theoretical mass of the peptide and the observed mass of the peptide was calculated using PEPTIDEMASS, an online tool by ExPasy (36), to identify the oxidative modification.

## Data availability

Raw MS data have been deposited as a MassIVE dataset with the Center for Computational Mass Spectrometry (ftp://massive.ucsd.edu/MSV000088593/).

## Supporting information

This article contains supporting information.

## Conflict of interest

The authors declare that they have no conflicts of interest with the contents of this article.
