# Supplementary material for: Escherichia coli alanyl-tRNA synthetase maintains proofreading activity and translational accuracy under oxidative stress
Source: J Biol Chem. 2022 Jan 20;298(3):101601. doi: 10.1016/j.jbc.2022.101601 (PMC8857464; doi:10.1016/j.jbc.2022.101601)
Supplement: Supplemental Figure S1 [file mmc3.pdf]

Figure S1

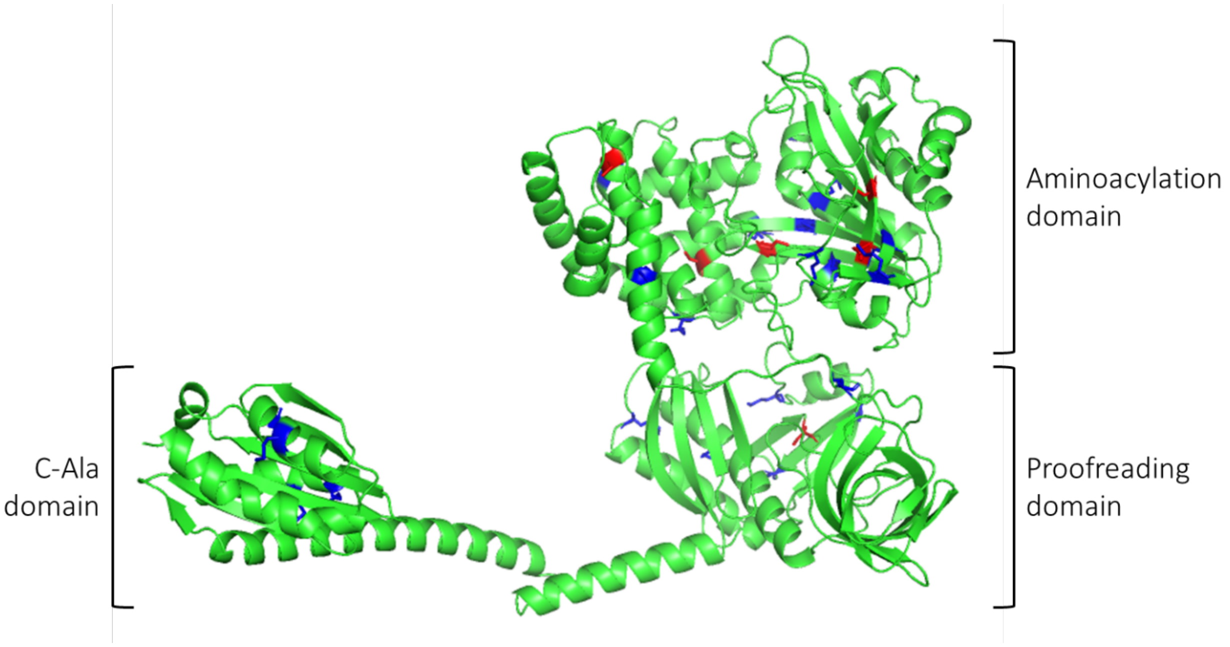

Figure S1. Alpha fold prediction of *E. coli* AlaRS depicting all 21 methionine residues (in blue) and all 6 cysteine residue (in red)
