# Supplementary material for: Escherichia coli alanyl-tRNA synthetase maintains proofreading activity and translational accuracy under oxidative stress
Source: J Biol Chem. 2022 Jan 20;298(3):101601. doi: 10.1016/j.jbc.2022.101601 (PMC8857464; doi:10.1016/j.jbc.2022.101601)
Supplement: Supplemental Figure S2 [file mmc4.pdf]

Figure S2

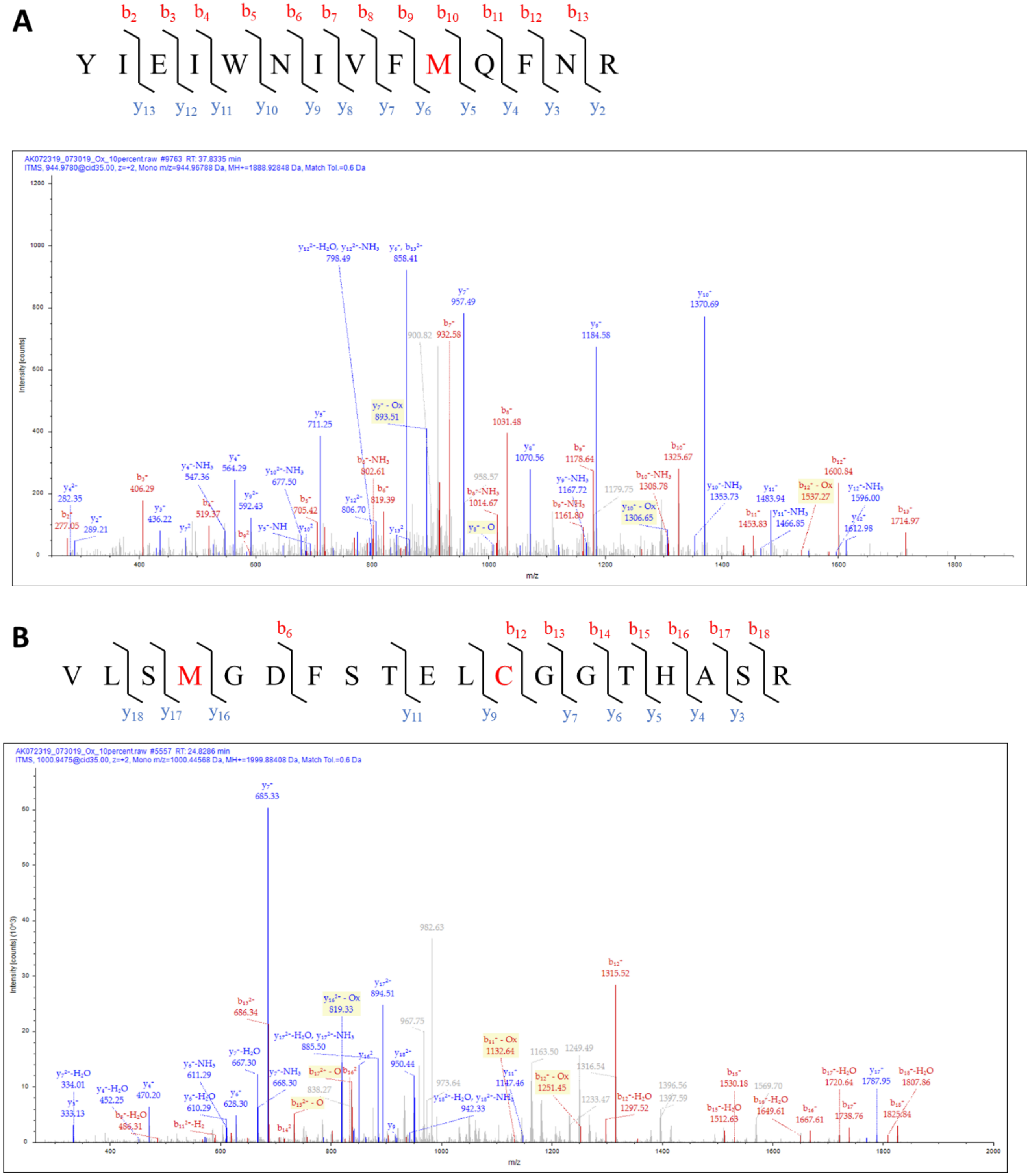

A
Y I E I W N I V F M Q F N R
AK072319_073019_Ox_10percent.raw #9763 RT: 37.8335 min
ITMS, 944.9780@cid35.00, z=+2, Mono m/z=944.96788 Da, MH+=1888.92848 Da, Match Tol.=0.6 Da
Intensity [counts]
m/z
B
V L S M G D F S T E L C G G T H A S R
AK072319_073019_Ox_10percent.raw #5557 RT: 24.8286 min
ITMS, 1000.9475@cid35.00, z=+2, Mono m/z=1000.44568 Da, MH+=1999.88408 Da, Match Tol.=0.6 Da
Intensity [counts] (10^3)
m/z

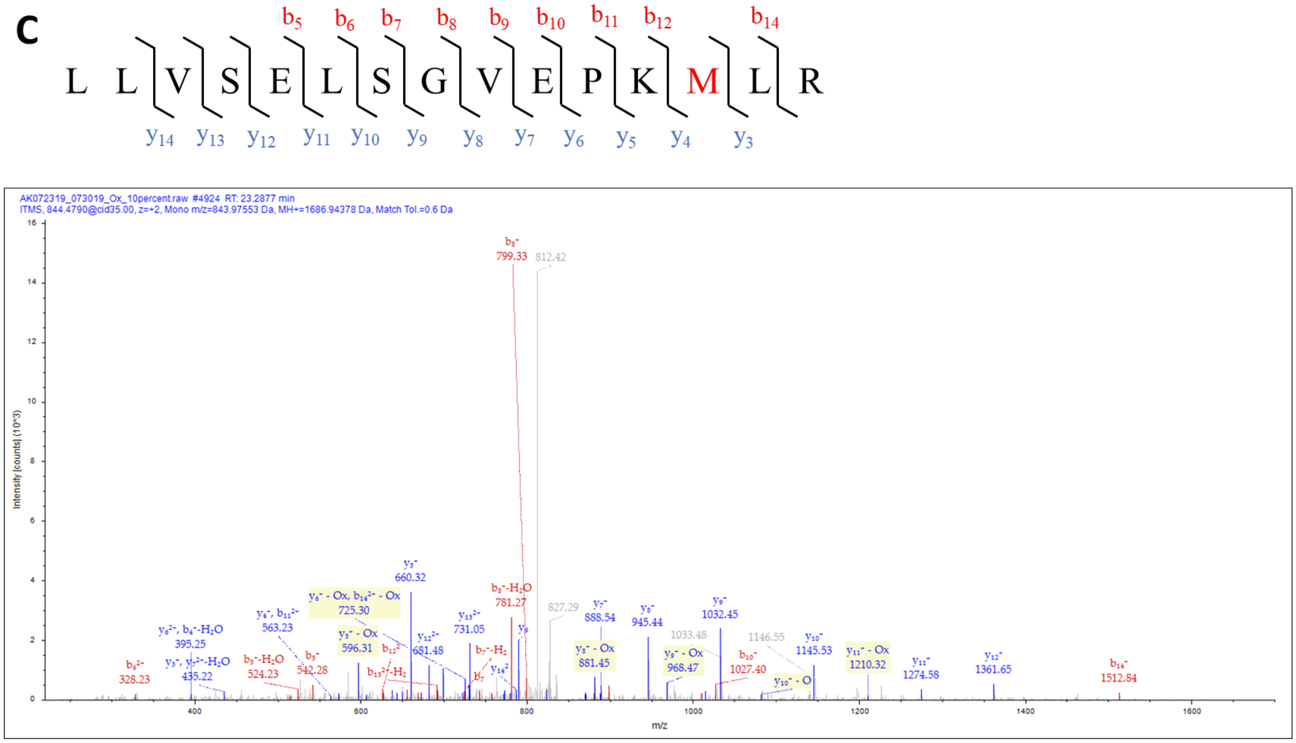

Figure S2. MS/MS spectra of *E. coli* AlaRS peptide upon treatment with H_2O_2. The MS/MS spectra depicts the fragmentation patterns of the peptides containing (A) oxidized Met217 residue (B) oxidized Met658 and oxidized Cys666 residues (C) oxidized M785 residue.
